# Developing a community-responsive research model in the healthcare system: a mixed-method study

**DOI:** 10.1186/s12874-024-02250-1

**Published:** 2024-05-25

**Authors:** Hooman Khanpoor, Mohammad Amerzadeh, Ahad Alizadeh, Omid Khosravizadeh, Sima Rafiei

**Affiliations:** 1 Student Research Committee, School of Public Health, Qazvin University of Medical Sciences, Qazvin, Iran; 2 Social Determinants of Health Research Center, Research Institute for Prevention of Non- Communicable Diseases, Qazvin University of Medical Sciences, Qazvin, Iran

**Keywords:** Responsiveness, Health system, Research, Structural equation modelling

## Abstract

**Background:**

Responsiveness to the population’s non-clinical needs encompasses various dimensions, including responsive research and an educational outreach plan at the community level. This study aims to develop a community-responsive research model in the healthcare system to ensure the connection between community-identified health priorities and research funds, as well as capacity-building efforts.

**Methods:**

A mixed-methods research study was conducted in three main phases, including a comprehensive literature review, a qualitative analysis of an expert panel’s points of view, and the developing of a model using the Equation Modeling (SEM) technique. R software version 3.2.4 was used to conduct statistical analysis, considering a significance level of 0.05.

**Results:**

Based on the literature review, 41 responsiveness components were identified from sixteen relevant studies conducted between 2000 and 2022. Ten sub-themes in four major themes, including planning, implementation, monitoring and evaluation, and action, were identified through qualitative content analysis. Standardized coefficients revealed that components such as dissemination of results to all stakeholders, research prioritization aligned with community needs, commitment to implement research findings, and collaborative learning had statistically significant effects on the community-responsive research model.

**Conclusion:**

It is essential to identify community health priorities by following a community-focused, priority-setting process based on the principles of community engagement to develop a community-responsive research model. Afterward, dissemination of research findings to all stakeholders, commitment to apply the obtained results in the real world, and promotion of shared learning among research partners have been proven to facilitate collaborative investigation and mutual understanding between the community and academic partners.

## Introduction

According to a report by the World Health Organization (WHO), health systems have three fundamental objectives: improving the community’s health condition, responding to people’s expectations, and ensuring financial fairness [1]. One of the most important goals is responsiveness to non-medical needs, which includes various dimensions, such as responsiveness to non-clinical expectations of people, responsiveness to public health services, and responsiveness in community-based education and community health applied research [2].

Regarding community-responsive research, the critical area of responsiveness is achieving a proper understanding of the capabilities, competitive advantages, available resources, strengths, and weaknesses of the community in conducting applied research in health domains, as well as identifying the unmet health and healthcare needs of a population [3, 4]. Some researchers believe that factors such as lack of time, insufficient skills in conducting research, lack of financial support, budget, and resource-related problems, lack of interest and motivation, failure to utilize research findings, and difficulties in statistical analysis are among the significant obstacles that impede the responsiveness of medical research [3, 4]. However, some more crucial factors, such as limited information about effective interventions to improve the community’s health outcomes, are not well represented in scientific research [5]. A community-academic partnership can identify health priorities, search for mutual benefits for all stakeholders, and use the obtained information to fill the gaps in community research efforts to tackle the issue [6, 7].

As the International Medical Sciences Association has emphasized, before conducting research in a population or community with limited resources, researchers need to make every effort to ensure that the research meets the health needs and priorities of the community [4]. Accordingly, the success of community-based research (CBR) depends on various factors, including the empowerment of community members to fulfill the role of researchers through engaging and maintaining partnerships with research organizations to determine community health priorities and disseminate the obtained results [8]. This approach will foster trusting research relationships and give community members an insight into new ways of approaching health [9]. To this end, CBR initiatives should develop an effective educational program that provides a core set of research-focused training to community members at the initial stage of the project to enhance their health literacy. Such an approach enables community members to contribute effectively during the research design and data collection processes. Furthermore, it is essential to ensure continuous and equitable training and guidance throughout the project, ensuring that diverse community members with different backgrounds and research skills can meaningfully participate in their expected roles as researchers [10]. This concept encompasses extensive public participation, ranging from health needs assessment and planning to implementation of health policies within the healthcare system and participation in financing, monitoring, and continuous evaluation [10]. Through this approach, the primary components of community-based research include fair participation, dedicated efforts to capacity building, particularly in research education, meaningful engagement of community members, particularly vulnerable and marginalized individuals, ineffective collaboration processes, and shared ownership [11]. Overall, the benefits of CBR lie in prioritizing research agendas and addressing research questions that reflect community needs, which can positively and influentially impact health policies [12].

Recently, funders have realized that community engagement increases the effectiveness of research projects and mentioned it as a demanding factor in translating results into community health benefits [13–15]. On this basis, research institutions should build supportive infrastructure and develop community research capacities to incentivize funding in research areas that bring about meaningful outcomes for communities [16]. Moreover, mutual trust, transparency, and awareness are essential to building effective partnerships, particularly in multilevel collaborations. Likewise, sustaining training programs for effective dissemination of research findings and finding funding opportunities were mentioned as requirements for addressing research training gaps, focusing on the identified needs in the future [16]. A recent Global Consortium of Knowledge for Change (K4C) by UNESCO on Community-Based Research has been developing research hubs through a participatory approach to bring academia together with the local community and produce new knowledge about the issues of the highest relevance to the community. Thus, there is a significant need for medical universities and health faculties to strengthen their community-based teaching to effectively convey the concept of community-based participatory research to students and academic staff [17].

The use of participatory methods in determining the contributing factors to the responsiveness of healthcare systems in terms of community-based research can result in interconnected variables being used in formulating a comprehensive model [18]. Thus, in the current study, we developed a community-responsive research model using different stakeholders’ points of view and a mixed-method research method building upon existing theoretical frameworks. The study aimed to design a responsive healthcare system model by identifying the influential dimensions of responsiveness in health research based on a comprehensive review and expert panel opinions.

## Materials and methods

### Study design and participants

This was a mixed-methods study, including a literature review and qualitative method to develop an initial community-responsive research model. First, a literature review was conducted to identify the different domains of responsiveness in health research. Next, an expert panel consisting of diverse stakeholders and informed people finalized the domains and subdomains of the responsive model. In the last phase, Confirmatory Factor Analysis (CFA) was used to examine the relationships between different extracted variables and develop the final model.

### Different phases of the study

First, we searched relevant keywords, including community-based participatory research, responsiveness model, health system, participatory research, and influencing factors in databases, such as Web of Science, Scopus, Google Scholar, OVID, EBSCO, SID, and Irandoc. Based on the study objectives, relevant information was extracted and compiled in a table format to depict the study title, publication date, methodology, and main findings.

In the second phase, to identify the influencing factors on the community-responsive research model, we presented an initial draft of features extracted from the literature review to the expert panel. Then, based on their understanding of the subject and the opinions of the expert panel, the critical components remained in the conceptual model. Furthermore, the expert panel members assessed the features to ensure their transparency, applicability, and compatibility with the country’s conditions. At the end of the panel, some of the features were removed or amended to improve the suitability and representativeness of the model. This phase of the study comprised five main phases: (1) familiarizing oneself with data; (2) generating codes; (3) developing themes; (4) reviewing and defining themes; and (5) producing the report [19, 20]. By prolonging engagement in data, participants developed potential codes and themes using a coding framework and peer debriefing. Then, through researcher triangulation, we diagramed theme connections to reach team consensus. Finally, after describing the extracted codes, we developed a conceptual framework and conceptualized different components within their relevant dimensions. The expert panel members were informed people with sufficient knowledge and experience in community-based participatory research, as well as academics, researchers, and policymakers in healthcare management, community medicine, community needs assessment, community advisory boards, and community health workers. Table 1 depicts the composition of the expert panel.

**Table 1:** The composition of the expert panel participants

Characteristics		Count (*n*)	Distribution (%)
Gender	Male	5	38.4
	Female	8	61.6
Age	30–40	3	23.07
	40–50	6	46.1
	≥ 50	4	30.83
Job title	Researcher	5	38.9
	Academics	4	30.7
	Policy makers	1	7.6
	Community advisory board member	1	7.6
	Community medicine member	1	7.6
	Health workers	1	7.6
Length of service	< 10	2	15.3
	10–20	6	46.1
	≥ 20	5	38.6

We developed a questionnaire in the qualitative phase, using literature review data and the experts’ opinions. The first part of the questionnaire included demographic information, and the second part involved the features of a responsive model with ten questions in 4 dimensions: planning, implementation, monitoring and evaluation, and action. Using a five-point Likert scale (from very low = 1 to very high = 5), study participants were asked to scale each factor based on their importance. For this purpose, some academics with sufficient expertise in conducting applied research and several health authorities with the necessary knowledge and experience in health research policy-making at the university level were invited to participate in the study and evaluate the importance of different factors on the responsive model. An appropriate sampling method in studies that apply SEM is 5 to 20 times greater than the number of components (10*15 = 150). After considering an attrition rate of 40%, a final sample size of 210 was achieved. The CFA technique was used in five stages, including formulating an initial model, data collection, establishment of consistent parameters, evaluation of the model fit, and interpretation of the model to examine the relationships between variables and present the final responsiveness model.

### Data analysis

In the qualitative phase, content analysis was used to determine the themes or concepts within the qualitative data. Then, to verify the factor structure of a set of variables and test the relationships between observed variables and their underlying latent constructs CFA was employed in R software version 3.2.4, with a significance level of 0.05.

## Results

The findings indicate that the components of responsiveness in the research, based on a comprehensive review of studies from 2000 to 2022, as identified in Table 2, are community participation in research and development, enhancement of shared learning among research partners, and prioritization of research areas aligned with needs. These components showed the highest frequency and occurrence in the context of healthcare responsiveness in the research, as evidenced by the analysis of 16 selected articles.

**Table 2:** The components extracted from the literature review

Components	
1. Collaborative learning among research partners 2. Balance between research and service delivery 3. Research prioritization aligned with the community needs 4. Community participation in research and development 5. Achievement of research indicators aligned with community needs 6. Dissemination of results to all stakeholders 7. Commitment to project evaluation and focusing on the main objectives of the project 8. Focus on community resources and capabilities 9. Community as a social identity 10. Interaction between students, academics, and community-based organizations 11. Transfer of knowledge, skills, and methods between communities and individuals 12. Health equity 13. Recognition of the value and legitimacy of experiential knowledge of research participants 14. Joint decision-making throughout the research process 15. Collective ownership of the knowledge generated by researchers 16. Participation of all members of the community 17. Provision of feedback to the community 18. Protect community interests 19. Respect to communities in case of biomedical research 20. Informed consent 21. Focus on the strengths and resources within the community 22. Facilitation of joint participation in all stages of the research	23. Integrate knowledge and action for the mutual interests of all partners 24. Promote a collaborative learning process and empowerment of community members to address social inequalities 25. Emphasize the cyclical nature of the research process 26. Consider health from a positive environmental perspective 27. Disseminate findings to all stakeholders 28. Democratize knowledge by validating multiple sources of knowledge and promoting the use of diverse dissemination methods 29. Social action for achieving social change and justice 30. Transparency 31. Adherence to ethics, law and regulations 32. Honesty 33. Impartiality and independence 34. Effectiveness, and efficiency 35. Continuous improvement of quality 36. Sustainable relationships and commitments 37. Development of knowledge, capacity, and values 38. Budget continuity 39. Programs and policy changes 40. Long-term capacity development 41. Continuous funding to support various stages of the project

### Conceptualization of components and related dimensions

**Fig. 1:**
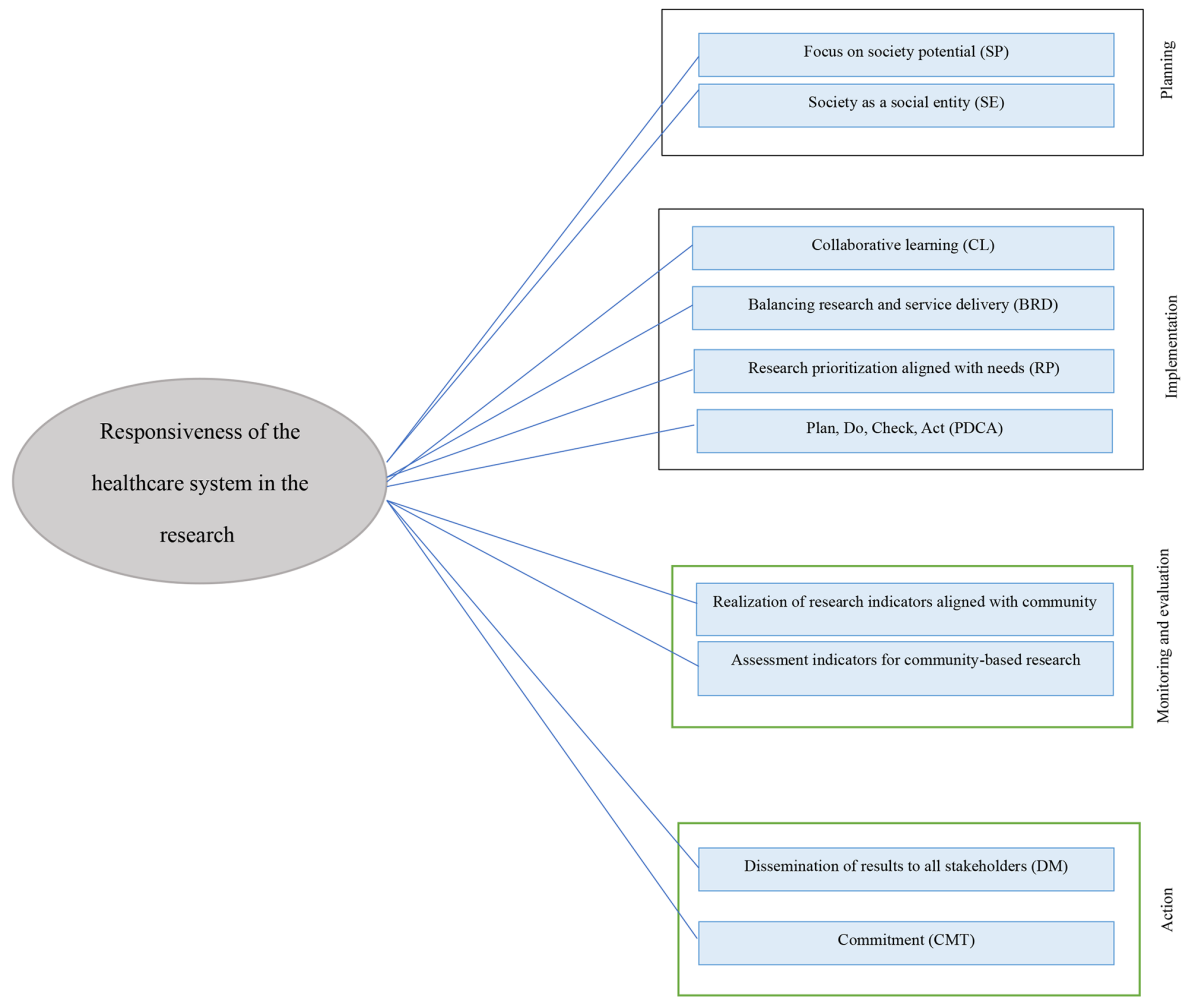
The conceptual model of responsiveness in the research healthcare system in the research domain

The influencing factors on research responsive model in the health system were organized in the form of a conceptual model which is shown in Fig. 1.

### The effects of different components on the responsiveness of the model

**Fig. 2:**
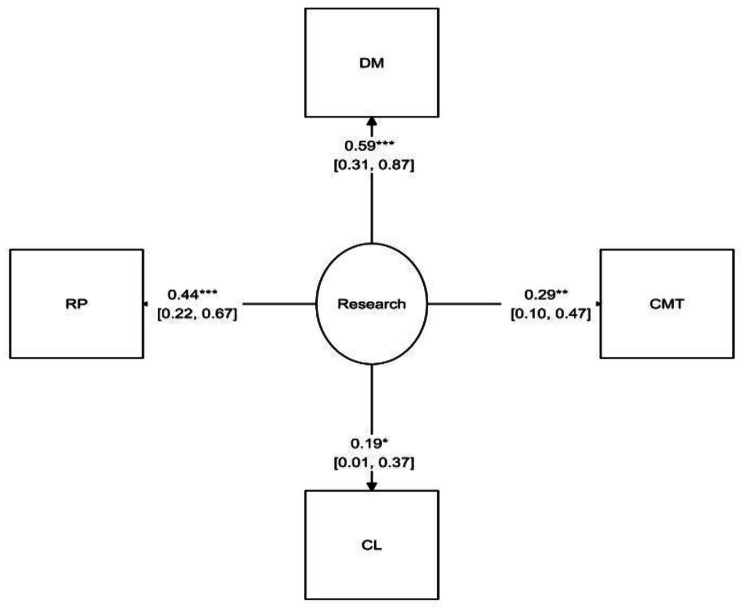
The impact of different components on the responsiveness model

Based on standardized coefficients depicted in Fig. 2; Table 3, dissemination of research results to all stakeholders, research prioritization aligned with needs, commitment, and collaborative learning had statistically significant positive effects on the health system’s responsiveness in community-based research. An increase of one standard deviation in each of the mentioned components would lead to a significant increase in the responsiveness of the healthcare system in the research domain.

**Table 3:** The impact of components on the responsiveness of community-based research system

Component	Non- standardized coefficient	Standard deviation	% confidence interval 95		Standard coefficient	*P*-value
			Upper bound	Lower bound		
Prioritizing research aligned with community needs	1	0	1	1	0.444	-
Dissemination of results to all stakeholders	1.642	0.753	3.117	0.166	0.591	0.029
Collaborative learning	0.412	0.226	0.855	0.032-	0.186	0.069
Commitment	0.863	0.35	1.549	0.177	0.285	0.014

## Discussion

This study aimed to develop a community-responsive research model in a healthcare system through the identification of influencing dimensions of responsiveness based on a literature review and expert panel opinions. Findings revealed that the influential components of responsiveness in the research domain could be classified into four main groups of planning, implementation, monitoring and evaluation, and collaborative action.

The first component emphasizes the significance of considering societal factors in research projects. This component encompasses two key aspects: focusing on society’s potential and recognizing society as a complex social entity. Regarding society’s capacity, there is a necessity to leverage the collective potential of all community members to identify research areas based on the actual needs of people. Likewise, several studies underscored the importance of engaging diverse stakeholders, such as community members, policymakers, and industry representatives, in the research process particularly in defining research subjects and evaluating their impacts on the society [21]. By involving these stakeholders from the outset, researchers can better understand the societal context, identify health needs, and develop practical solutions that are more likely to be accepted and implemented. Furthermore, focusing on society’s potential can foster collaborative partnerships and facilitate the co-creation of knowledge, expertise, and intellectual resources as well as empowering physical, and monetary infrastructure [22]. Such collaborations can also enhance the relevance and applicability of research findings while endorsing sustainable positive change in the society’s condition [23].

The second aspect considers society as a social entity which is not merely a collection of individuals but a complex entity with intricate dynamics, norms, and power structures. In fact, research works in the field of health topics must acknowledge and navigate these societal complexities to effectively address health issues [24]. Researchers need to consider social determinants of health, such as socioeconomic status, education, and cultural factors, which can significantly influence health outcomes [25]. By adopting a societal lens, researchers can better understand the root causes of health disparities and develop interventions that address systemic barriers and promote health equity [24]. Moreover, recognizing society as a social entity necessitates a critical examination of power dynamics and the potential for research to perpetuate or challenge existing inequalities [26]. Participatory approaches that engage marginalized communities and prioritize their voices can also help mitigate power imbalances and ensure that research efforts align with societal needs and values [21]]. In conclusion, the planning component underscores the importance of considering societal factors from the outset. In fact, by focusing on society’s potential and acknowledging society as a complex social entity, researchers can develop more relevant, impactful, and equitable health solutions.

The second component underscores the importance of collaborative and iterative processes in health research. This factor encompasses four key aspects including: collaborative learning, balancing research and service delivery, research prioritization aligned with the community’s needs, and the Plan, Do, Check, Act (PDCA) cycle. Regarding collaborative learning among research partners, effective implementation requires collaboration among diverse stakeholders, such as researchers, practitioners, policymakers, and community members [21]. Such collaboration fosters mutual understanding, shared decision-making, and the co-creation of knowledge [22]. It also enhances the relevance and applicability of findings while building capacity and empowering stakeholders [23]. Balancing research and service delivery addresses the tension between pursuing new knowledge and providing immediate services or interventions [23]. Effective implementation requires an approach that integrates research and service delivery, allowing researchers to address community needs while advancing scientific understanding [21]. Research prioritization aligned with the community’s needs is another crucial aspect. Accordingly, a responsive health research must prioritize research questions and activities that are congruent with the identified needs of communities. Involving stakeholders in the prioritization process ensures that efforts address relevant and pressing health issues, increasing the potential for meaningful impact [24]. Finally, the PDCA cycle, also known as the Deming cycle, is a recognized approach to continuous improvement [25]. In health research implementation domain, the PDCA cycle involves planning activities, executing the plan, monitoring and evaluating processes, outcome evaluation and making necessary adjustments based on obtained findings. This iterative approach promotes ongoing learning, adaptation, and refinement which consequently enhance the responsiveness and effectiveness of health research efforts [26]. In conclusion, the implementation component focuses on collaborative processes, balancing research and service delivery, aligning research priorities with identified needs, and embracing a continuous improvement mindset through the PDCA cycle. Incorporating these aspects can enhance the relevance, impact, and sustainability of health research, contributing to improved health outcomes and societal well-being.

The third component, monitoring and evaluation highlights the importance of outcome evaluation and the alignment of health research efforts with community needs. This component encompasses two key aspects including realizing research indicators in line with community needs and assessing indicators to develop community-based research projects. Regarding the realization of research indicators associated with community needs, effective monitoring and evaluation requires the identification of relevant indicators and subsequent tracking of them to ensure that research outcomes are consistent with the community’s identified needs [31] .These indicators should be co-developed with different stakeholders to certify their relevance and meaningfulness [27]. By monitoring the realization of these indicators, researchers can assess the degree to which their work addresses the priorities and concerns of the community, enabling them to make necessary adjustments and enhance the responsiveness of their research efforts [21]. The assessment indicators for community-based research involve developing and monitoring indicators that assess the quality and effectiveness of community-based research processes [27]. These indicators may include measures of community engagement, capacity building, power-sharing, and sustainability [28]. By assessing these indicators, researchers can evaluate the extent to which their research adheres to the principles of community-based participatory research (CBPR) and addresses issues of equity, empowerment, and social justice [29]. Furthermore, monitoring and evaluation based on these indicators can inform the refinement of research processes, strengthen community partnerships, and enhance the overall responsiveness and impact of health research initiatives [11]. To sum up, the monitoring and evaluation component highlights assessing the degree of consistency between research outcomes and community needs as well as the level of quality community-based research processes that have been achieved. By incorporating these aspects, researchers can ensure that their efforts are responsive and contribute to positive and sustainable change in the community they serve.

The fourth component which is collaborative action underscores the importance of translating research findings into tangible outcomes and ensuring their effective dissemination to relevant stakeholders. This component involves two key aspects: dissemination of results to all stakeholders and commitment. Regarding the former aspect, effective dissemination is crucial for maximizing the impact and responsiveness of health research [27]. This process should involve sharing findings with diverse stakeholders, including community members, policymakers, practitioners, and researchers. Dissemination strategies should be tailored to different stakeholder groups’ needs and preferences, employing various formats and channels like community forums, policy briefs, academic publications, and multimedia platforms. Effective dissemination increases the visibility and accessibility of findings while fostering dialogue, collaboration, and the co-creation of knowledge [21].

The commitment highlights the need for sustained commitment from all stakeholders to ensure the successful translation of research findings into action. This commitment involves dedicating resources, capacity building, and establishing mechanisms for ongoing collaboration and knowledge exchange [30]. Commitment from researchers entails a willingness to engage in long-term partnerships, adapt research processes based on community feedback, and advocate for implementing evidence-based interventions. Furthermore, commitment from community stakeholders involves active participation in research activities, providing guidance and feedback, and taking ownership of research outcomes. Moreover, commitment from policymakers and decision-makers is essential for translating research findings into policies and practices that can drive systemic change and improve population health outcomes [31]. To put it briefly, the action component emphasizes the importance of disseminating research findings to all stakeholders and fostering a sustained commitment to translating these findings into tangible outcomes. By incorporating these aspects, researchers can enhance their work impact, relevance, and sustainability, ultimately contributing to positive and lasting change in the communities they serve.

While the study identified essential components of community-responsive research model in the health sector through a comprehensive literature review and expert panel, there are some limitations to consider. First, selection bias might have been happened during the process of selecting the members of an expert panel. Second, the generalizability of the identified components could be limited, as they might be influenced by the specific contexts or disciplines represented in the reviewed literature and the expert panel. Therefore, the applicability of these components across different health research domains, geographical regions, or cultural settings may vary. Third, responsiveness in health research is a dynamic and evolving concept. Thus, the identified components, while relevant at the time of the study, may require periodic re-evaluation and updating to remain aligned with emerging trends, methodologies, or societal needs. To address these potential limitations, future research could expand the scope of the review, employ more inclusive and representative selection processes, explore the generalizability and practical implementation of the identified components, and actively engage a diverse range of stakeholders in refining and validating the findings.

## Conclusion

This study has identified four crucial components that underpin responsive health research: planning, implementation, monitoring and evaluation, and action. These components underscore the significance of adopting a comprehensive and participatory approach that involves diverse stakeholders throughout the research process. In conclusion, responsive health research is a multifaceted endeavor that requires a holistic approach encompassing careful planning, collaborative implementation, rigorous monitoring and evaluation, and a commitment to action. By embracing these components, researchers can enhance their work relevance, impact, and sustainability, ultimately contributing to improved health outcomes and societal well-being.

## Data Availability

The datasets used and/or analyzed during the current study available from the corresponding author on reasonable request. The entire dataset is in Farsi language. The Data can be available in English language for the readers and make available from the corresponding author on reasonable request.
